# S172. BRAIN METABOLITES AND THE RELATION WITH COGNITION AND PSYCHOTIC SYMPTOMS IN MEDICATION-FREE PSYCHOSIS AND CONTROLS: A PHARMACOLOGICAL MAGNETIC RESONANCE SPECTROSCOPY STUDY

**DOI:** 10.1093/schbul/sby018.959

**Published:** 2018-04-01

**Authors:** Claudia Vingerhoets, Geor Bakker, Marieke van der Pluijm, Oswald Bloemen, Liesbeth Reneman, Matthan Caan, Jan Booij, Therese van Amelsvoort

**Affiliations:** 1 Maastricht University; 2 Academic Medical Center; 3 GGz Centraal,Maastricht University; 4 Academic Medical Center, University of Amsterdam

## Abstract

**Background:**

Psychotic disorders are complex neuropsychiatric disorders characterized by positive, negative and cognitive symptoms. Over the recent years, several neurotransmitter systems and neurometabolites have been related to psychotic disorders but the exact underlying neurobiological mechanisms are still not well understood. One neurotransmitter system that has been increasingly related to psychosis is the cholinergic muscarinic system. Increased choline concentrations and reduced muscarinic M1 receptor expression have been reported in schizophrenia. Therefore, the present study investigated brain metabolite concentrations, their responsivity to M1 receptor blockage, and their relation to cognitive, positive and negative symptoms in psychosis.

**Methods:**

31 medication-free subjects with a psychotic disorder (mean age 27 years) and 31 gender, age and IQ-matched healthy control subjects (mean age 25 years) were enrolled in the study. 1H-proton magnetic resonance spectroscopy (1H-MRS, PRESS) was used to measure brain metabolites in the anterior cingulate cortex (ACC) and striatum. Metabolites measured included choline (Cho), glutamate (Glu), glutamine (Gln), GLX, myoinositol (MI), N-acetylaspartate (NAA) and gluthatione (GSH) (metabolite to creatine ratios were analyzed). All subjects were measured twice: once after placebo and once after a pharmacological challenge (4 mg. biperiden, a M1 receptor antagonist). The order of drug – challenge was counterbalanced. In addition, cognitive function was assessed using the Cambridge Neuropsychological Test Automated Battery (CANTAB) and psychotic symptom severity was assessed with the Positive and Negative Syndrome Scale (PANSS) to examine the relation between brain metabolites and cognition and psychosis symptoms.

**Results:**

No significant differences were found in both ACC and striatal brain metabolite levels between subjects with a psychotic disorder and controls after placebo. Moreover, M1 blockade did not significantly affect brain metabolite levels in these regions and no group x challenge interaction effects were found. In addition, in both groups, no correlation was found between cognitive functioning and any of the brain metabolites. In subjects with a psychotic disorder, a positive correlation was found between striatal choline levels (after placebo) and negative symptom severity (p = 0.024).

**Discussion:**

These results suggest that there are no differences in ACC and striatal brain metabolites between medication-free subjects with a psychotic disorder and healthy controls and that these metabolites are not influences by acute muscarinic M1 receptor antagonism. The significant correlation between striatal choline and negative symptom severity in the psychosis group could indicate that the cholinergic system is involved in negative symptom pathology. This is the first study that examined the influence of M1 receptor blockade on brain metabolites and therefore these results warrant replication.

